# A Novel Improved Feature Extraction Technique for Ship-Radiated Noise Based on IITD and MDE

**DOI:** 10.3390/e21121215

**Published:** 2019-12-12

**Authors:** Zhaoxi Li, Yaan Li, Kai Zhang, Jianli Guo

**Affiliations:** 1 School of Marine Science and Technology, Northwestern Polytechnical University, Xi′an 710072, China; lizhaoxi@mail.nwpu.edu.cn; 2 Department of Computer and Information of Science and Engineering, University of Florida, Gainesville, FL 32611, USA; zhangkai6@ufl.edu; 3 School of Mathematics and Computer Science, Shaanxi University of Technology, Hanzhong 723001, China; gk2814@163.com

**Keywords:** ship-radiated noise, multiscale dispersion entropy(MDE), improved intrinsic time-scale decomposition (IITD), intrinsic scale component (ISC), feature extraction

## Abstract

Ship-radiated noise signal has a lot of nonlinear, non-Gaussian, and nonstationary information characteristics, which can reflect the important signs of ship performance. This paper proposes a novel feature extraction technique for ship-radiated noise based on improved intrinsic time-scale decomposition (IITD) and multiscale dispersion entropy (MDE). The proposed feature extraction technique is named IITD-MDE. First, IITD is applied to decompose the ship-radiated noise signal into a series of intrinsic scale components (ISCs). Then, we select the ISC with the main information through the correlation analysis, and calculate the MDE value as feature vectors. Finally, the feature vectors are input into the support vector machine (SVM) for ship classification. The experimental results indicate that the recognition rate of the proposed technique reaches 86% accuracy. Therefore, compared with the other feature extraction methods, the proposed method provides a new solution for classifying different types of ships effectively.

## 1. Introduction

During the development of passive sonar, the ship-radiated noise signal has been widely used in the detection, tracking, and classification of ship targets. As it contains a lot of information about ship characteristics, ship-radiated noise has always been a research hotspot of underwater acoustic signal processing. Hence, extracting effective and reliable ship-radiated noise characteristic parameters is highly valuable [1,2]. Ship-radiated noise signals usually have time-variant and nonstationary characteristics. Especially in the early stage of signal processing, the ship feature is weak and is completely drowned out by the complexity of marine environments [3,4]. Therefore, in order to realize the effective ship signal, suppressing the background noise and effects of aliasing between the feature information from the original signal is becoming in urgent need of solving.

Due to the rapid development of ship-radiated noise signal processing technology, some researchers have proposed many nonlinear and nonstationary signal processing methods for the feature extraction of underwater acoustic target signals, such as empirical mode decomposition (EMD) [5,6], intrinsic time-scale decomposition (ITD) [7,8], local mean decomposition (LMD) [9], and their improved algorithms [10,11,12,13,14]. Hong [15] proposed ensemble EMD (EEMD) and energy distribution to extract the energy difference, which is an efficient feature extraction technique for ship-radiated noise. Li [16] proposed an improved energy feature extraction technique for ship-radiated noise, which combined CEEMDAN and EE to extract the hybrid energy feature. Frei [17] proposed an adaptive time-frequency analysis method, which can decompose the nonstationary signal into a series of single component signals with the physical meanings of instantaneous frequencies. In recent decades, these studies have provided rich reference information, which is widely used in fault diagnosis [18,19], biomedicine [20,21], geophysics [22], and hydroacoustics [23,24]. Compared with the EMD method, the ITD method has obvious advantages in terms of computational efficiency and processing edge effects. However, the definition of the baseline of the ITD method is based on the linear transformation of the signal itself, and may cause a glitch and distortion of the proper rotation components obtained by the decomposition. Based on this, we used akima interpolation [25] to improve the ITD method; then, the IITD algorithm was proposed. Therefore, this is a feasible way to decompose the ship-radiated noise signal by IITD to extract effective ISCs.

Entropy theory can efficiently evaluate the complexity of the time series and reduce the dimension of the feature vector and fully represent the characteristics of the series. Hence, there are many methods for complexity measurement, including Shannon entropy [26], sample entropy (SampEn) [27,28], permutation entropy (PE) [29], and fuzzy entropy [30], which have been successfully applied in the field of fault diagnosis and the medical field. However, SampEn is time consuming for large data calculations and is susceptible to mutated signals. While the PE is faster, it fails to consider the mean value of amplitudes and differences between the amplitudes value. In order to overcome the drawbacks of SampEn and PE, a new measure of the complexity method, named dispersion entropy (DE), was proposed by Mostafa Rostaghi and Hamed Azami in 2016 [31]. The advantage of the DE algorithm is that the calculation speed is fast, the influence of the noisy signal is small, and it considers the influence of the magnitude relationship between amplitudes of the signal. Since all of the above methods are based on a single scale, they fail to account for the interrelationship of entropy and temporal scales. To remedy this, Costa etal. proposed the multiscale entropy (MSE) algorithm, in which scales are generated by the coarse-graining process [32]. The coarse-graining process has better stability in feature extraction and can be combined with arbitrary entropy estimators for multiscale analysis. Regarding this advantage, a multiscale dispersion entropy (MDE) procedure was put forward to estimate the complexity of the original time series over a range of scales [33]. Therefore, the MDE of the signal was adopted in this paper to identify the feature information of the ship-radiated noise signal.

In this paper, an effective feature extraction technique for ship-radiated noise via IITD and multiscale dispersion entropy (MDE) is introduced, named IITD-MDE. The proposed technique not only retains the advantages of existing techniques, but also overcomes the disadvantages of ITD and dispersion entropy (DE).

The rest of the paper is organized as follows: Section 2 first describes the ITD and DE algorithms, and based on this, the IITD and MDE algorithms are proposed. The IITD-MDE method flow is described in Section 3. The experiments are verified and analyzed by real ship-radiated noise datasets in Section 4. The conclusions are given in Section 5.

## 2. Theory of IITD-MDE

### 2.1. IITD Algorithm

The ITD method realizes the signal decomposed by the linear transformation method, which appears the obvious signal distortion from the second component. Therefore, first, this section explains the physical meaning of the ITD decomposed method, and then proposes an improved ITD method (IITD).

#### 2.1.1. ITD

Suppose $\left\{X_{t},t\geq 0\right\}$ is a real-valued signal, let $\left\{\tau_{k},k=1,2,\cdots \right\}$ denote the local extrema of $X_{t}$, and for convenience define $\tau_{0}=0$. we defined L as the baseline extraction operator for $X_{t}$, and $X_{t}$ can be decomposed as [17]:

(1) $$X_{t}=LX_{t}+(1-L)X_{t}=L_{t}+H_{t},$$

where, $L_{t}=LX_{t}$ is the baseline signal, and $H_{t}=(1-L)X_{t}$ is proper rotation component.

To simplify the notation, let $X_{k}$ and $L_{k}$ denote $X\left(\tau_{k}\right)$ and $L\left(\tau_{k}\right)$, respectively. Suppose that $L_{t}$ and $H_{t}$ have been defined on $\left[0,\tau_{k}\right]$, and $X_{t}$ is available for $\left[0,\tau_{k+2}\right]$. We can define L on the interval $\left[\tau_{k},\tau_{k+1}\right]$ between successive extrema as follows:

(2) $$LX_{t}=L_{k}+\left(\frac{L_{k+1}-L_{k}}{X_{k+1}-X_{k}}\right)\left(X_{t}-X_{k}\right),$$

(3) $$L_{k+1}=\alpha \left[X_{k}+\left(\frac{\tau_{k+1}-\tau_{k}}{\tau_{k+2}-\tau_{k}}\right)\left(X_{k+2}-X_{k}\right)\right]+(1-\alpha )X_{k+1},$$

where 0<α<1 is typically selected as α=1/2. We were able to define the proper rotation operator $H_{t}$ as

(4) $$HX_{t}=(1-L)X_{t}=H_{t}=X_{t}-L_{t},$$

Given that signal $X_{t}$ is decomposed, it can be expressed as

(5) $$X_{t}=HX_{t}+LX_{t}=HX_{t}+(H+L)LX_{t}=(H\sum_{k=0}^{p-1}L^{k}+L^{p})X_{t},$$

where $HL^{k}X_{t}$ is the (k+1)th proper rotation component (PRC) and $L^{p}X_{t}$ is the monotonic trend signal.

The ITD method obtained the baseline by linear transformation, which caused a glitch and distortion. Therefore, we present the IITD method, which replaces the linear transformation in the ITD method with akima interpolation. While akima interpolation is used, it is different from the envelope mean based on local extrema in EMD because IITD only requires one akima interpolation per decomposition.

#### 2.1.2. Comparison of Baseline-Fitting Method

**Figure 1:**
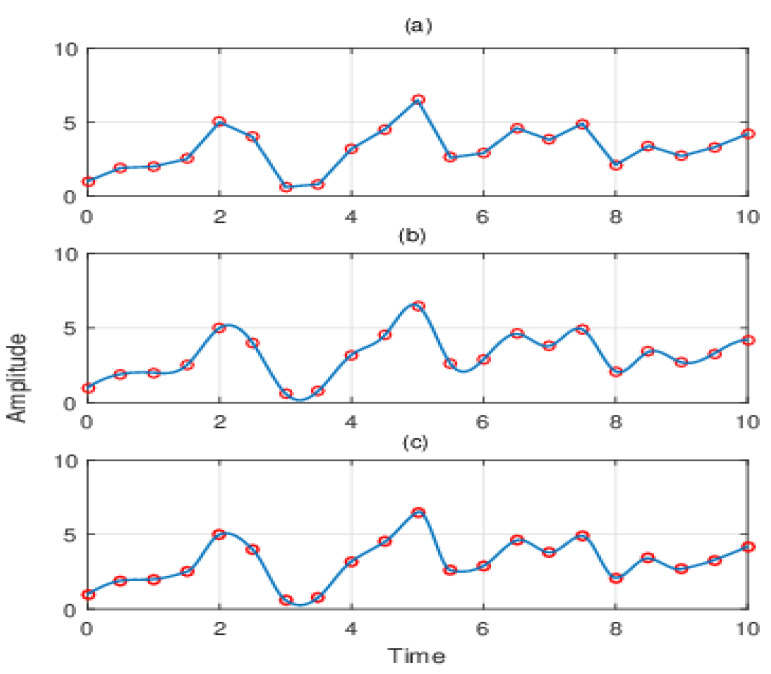
The comparison of the interpolation methods: (**a**) linear interpolation, (**b**) cubic spline interpolation, and (**c**) akima interpolation.

The comparison with the interpolation method is shown in Figure 1. The above three methods of the curve fitting method are used to interpolate discrete points, including linear interpolation, cubic spline interpolation, and akima interpolation. Figure 1a,b shows that the cubic spline interpolation has better smoothness and continuously differentiates second order interpolation rather than linear interpolation, but it will cause the phenomenon of “overshoot”. Therefore, the proposed method, combined with akima interpolation, can effectively avoid the overshoot and maintain the advantages of cubic spline interpolation. As shown in Figure 1c, this method has a better fitting effect, avoids the phenomenon of “overshoot”, and has better smoothness.

#### 2.1.3. Intrinsic Scale Component (ISC)

**Figure 2:**
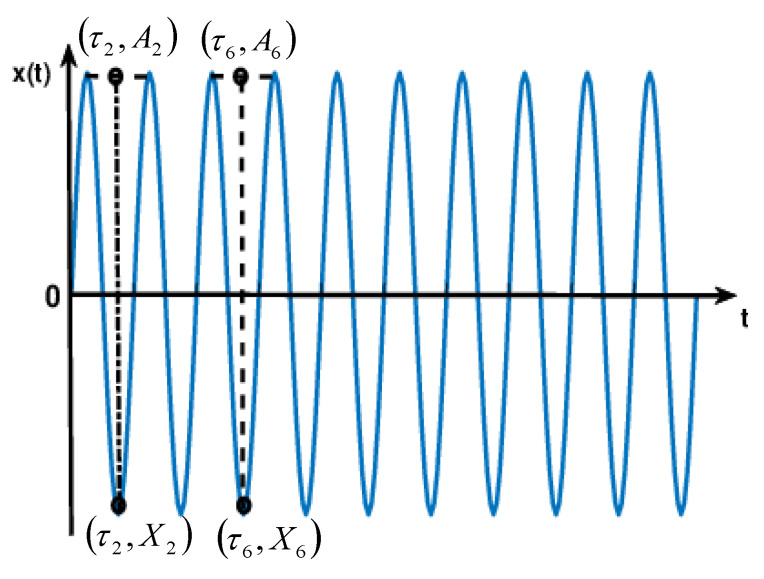
Intrinsic scale component (ISC) satisfies the conditions.

In the ITD method, PRC should satisfy the baseline signal control points $L_{k+1}=0$. Based on this, we defined the ISC of the physical meaning of instantaneous frequency and satisfied the conditions as follows:

(1) Any two adjacent maxima and minima are monotonic in the whole data segment.

(2) Let $\left\{\tau_{k}\;,\;k=1,2,\cdots ,M\right\}$ denote the local extrema of $\left\{X_{k}\;,\;k=1,2,\cdots ,M\right\}$, the line connected the maximum value at $\tau_{k}$ and $X_{k}$ and minima value at $\tau_{k+2}$ and $X_{k+2}$, the function value of extreme points $\left(\tau_{k,}X_{k+1}\right)$ at the corresponding time $\tau_{k+1}$ is $A_{k+1}=X_{k}+\left(\frac{\tau_{k+1}-\tau_{k}}{\tau_{k+2}-\tau_{k}}\right)\left(X_{k+2}-X_{k}\right)$ and its radio to $X_{k+1}$ remains the same. These are satisfied as follows: $\alpha \left[X_{k}+\left(\frac{\tau_{k+1}-\tau_{k}}{\tau_{k+2}-\tau_{k}}\right)\left(X_{k+2}-X_{k}\right)\right]+(1-\alpha )X_{k+1}=0$, $\frac{A_{2}}{X_{2}}=\cdots =\frac{A_{6}}{X_{6}}=\cdots \mu$, where α is typically chosen to be 0.5, any there is a choice of α in the interval (0,1). ISC satisfies the conditions, as shown in Figure 2.

#### 2.1.4. IITD

IITD is an algorithm for decomposing the physical signals into a collection of ISCs, which are independent of each other.

(1) Let $\left\{\tau_{k},k=1,2,\cdots \right\}$ denote the local extrema of $X_{t}$, and take the same steps as the ITD method’s Equations (2) and (3) to extract each baseline signal point $L_{k}$.

(2) Take the mirror symmetric extension method to process the $X_{t}$ in order to obtain the left extreme value at $\tau_{0}$ and $X_{0}$ and the right extreme value at $\tau_{M+1}$ and $X_{M+1}$. Define k=0 and k=M−1 respectively, according to Equations (1) and (2), and find the values of $L_{1}$ and $L_{M}$. Then, use akima interpolation to fit all the $L_{k}$ and get the baseline signal $L_{1}\left(t\right)$.

(3) Separate the baseline signal $L_{1}\left(t\right)$.

(6) $$h_{1}\left(t\right)=X_{t}-L_{1}\left(t\right),$$

Suppose baseline signal $L_{k+1}\neq 0$, then $h_{1}\left(t\right)=ISC_{1}$. If the baseline signal $L_{k+1}=0$, then set $h_{1}\left(t\right)$ as the original signal to repeat steps (1–2), loop k until $h_{1k}\left(t\right)=ISC_{1}$. Then, separate $ISC_{1}$ from the original signal as the new signal $r_{1}\left(t\right)$.

(4) Set $r_{1}\left(t\right)$ as a given signal, repeat steps (1–3) and $X_{t}$ can be decomposed as

(7) $$X_{t}=\sum_{n=1}^{n}ISC_{n}+r_{n}\left(t\right),$$

where $ISC_{n}$ is the nth intrinsic scale component (ISC), and $r_{n}\left(t\right)$ is a monotonic trend signal.

### 2.2. MDE Algorithm

#### 2.2.1. DE

(1) Considering a given nonlinear time series $x=\left\{x_{j},j=1,2,\cdots ,N\right\}$, the normal cumulative distribution function (NCDF) of x is calculated as follows:

(8) $$y_{j}=\frac{1}{\sigma \sqrt{2\pi }}\int_{-\infty }^{x_{j}}e^{\frac{-{\left(t-\mu \right)}^{2}}{2\sigma^{2}}}dt,$$

where μ and σ represent the mean and standard deviation of time series x, respectively.

(2) Then, map $y_{j}$ to $z_{j}$, by using the following definition:

(9) $$z_{j}^{}=round\left(c\cdot y_{j}+\frac{1}{2}\right),$$

where c is an integer.

(3) Time series $z_{i}^{m,c}$ is made according to

(10) $$z_{i}^{m,c}=\left\{z_{i}^{c},z_{i+d}^{c},\cdots ,z_{i+(m-1)d}^{c}\right\},\;i=1,2,\cdots ,N-(m-1)d,$$

Each time series $z_{i}^{m,c}$ is mapped to dispersion pattern $\pi_{v_{0}v_{1}\cdots v_{m-1}}$, where

(11) $$z_{i}^{c}=v_{0},z_{i+d}^{c}=v_{1},\cdots ,z_{i+(m-1)d}^{c}=v_{m-1},$$

(4) For each $c^{m}$ potential dispersion pattern $\pi_{v_{0}v_{1}\cdots v_{m-1}}$, its relative frequency is obtained as follows:

(12) $$p(\pi_{v_{0}v_{1}\cdots v_{m-1}})=\frac{\#\left\{i|\;i\leq N-(m-1)d,\;z_{i}^{m,c}\;has\;type\;\pi_{v_{0}\cdots v_{m-1}}\right\}}{N-(m-1)d},$$

In fact, $p\left(\pi_{v_{0}v_{1}^{}\cdots v_{m-1}}\right)$ shows embedding vector $z_{i}^{m,c}$ maps to the number of dispersion pattern $\pi_{v_{0}v_{1}\cdots v_{m-1}}$, divided by the total number of $z_{i}^{m,c}$.

(5) Finally, the DE value is calculated as follows:

(13) $$DE(x,m,c,d)=-\sum_{\pi =1}^{c^{m}}p(\pi_{v_{0}v_{1}\cdots v_{m-1}})\cdot In(p(\pi_{v_{0}v_{1}\cdots v_{m-1}})),$$

#### 2.2.2. MDE

**Figure 3:**
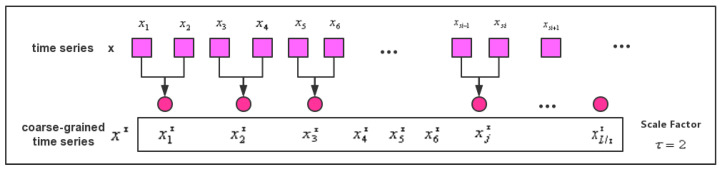
The coarse-grained process of MDE.

In order to solve the incomplete problem of extracting the complexity of the signal in the single scale, we propose the MDE method. It has better stability in the coarse-grained process and the advantage of feature extraction and error calculation of the signal. If scale factor τ=2, the coarse-grained process of MDE can be described, as in Figure 3. 

(1) Define a given time series {x(i), i=1,2,⋯,L}, $y^{\left(\tau \right)}$ that can be obtained by coarse-grained signal at scale factor τ:

(14) $$y_{j}^{\left(\tau \right)}=\frac{1}{\tau }\sum_{i=(j-1)\tau +1}^{j\tau }x(i),\;1\leq j\leq L/\tau ,$$

(2) Each coarse-grained series $y^{\left(\tau \right)}$ can be calculated by

(15) $$MDE(x,m,c,d,\tau )=DE(y^{\left(\tau \right)},m,c,d),$$

### 2.3. Comparison between ITD, IITD, and EMD

In order to compare ITD, IITD, and EMD, simulation signals are taken as

(16) $$\left\{ \begin{array}{l} x_{1}\left(t\right)=\cos(10\pi t)+\cos\left(50\pi t\right)\\ x_{2}\left(t\right)=randn(t)\\ x(t)=x_{1}(t)+x_{2}(t) \end{array}\right.,$$

where, x(t) consists of $x_{1}\left(t\right)$ with the sampling frequency of 1 kHz and standard Gaussian white noise $x_{2}\left(t\right)$.

**Figure 4:**
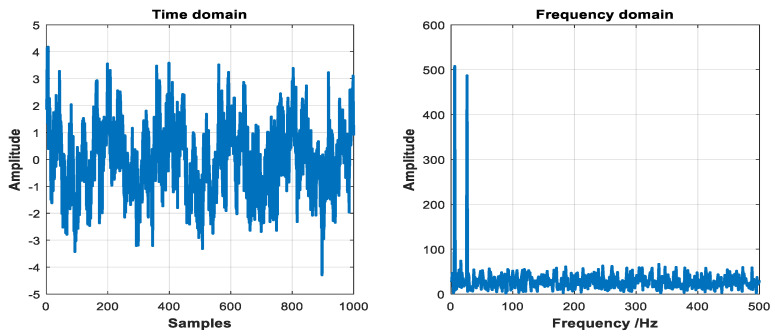
The time-frequency domain waveforms of x(t).

**Figure 5:**
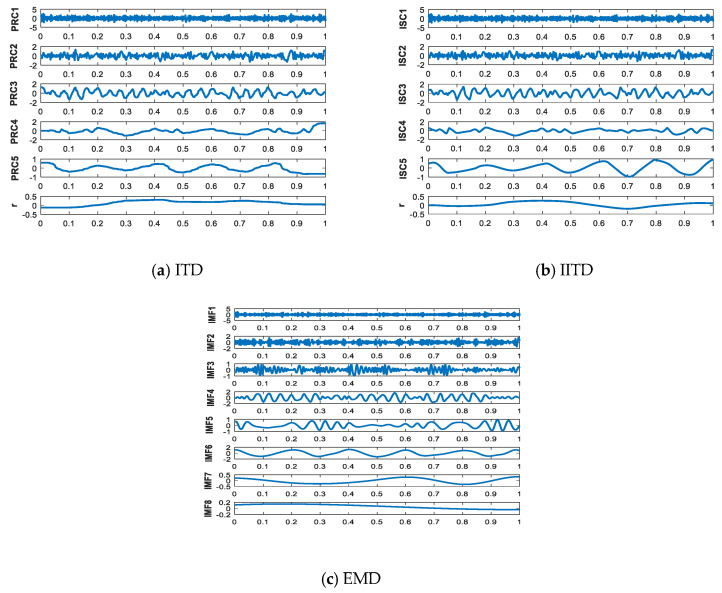
The results of decomposing.

The time-frequency domain waveforms of x(t) are shown in Figure 4. The results decomposed by ITD, IITD, and EMD are depicted in Figure 5. Compared with Figure 2 and Figure 3, the result of decomposed by ITD has obvious deformation and end effect. In addition, it can be seen from the monotonic trend of signal r that the fitting error of ITD decomposition is also relatively large. The IMFs of EMD decomposed appears illusive components and model aliasing phenomenon. Based on the above comparison, the original signal can be decomposed more accurately by using IITD method. At the same time, it can overcome the defect of model aliasing and illusive components by EMD method and waveform distortion caused by the ITD method.

### 2.4. Comparison between MSE, MPE, and MDE

**Figure 6:**
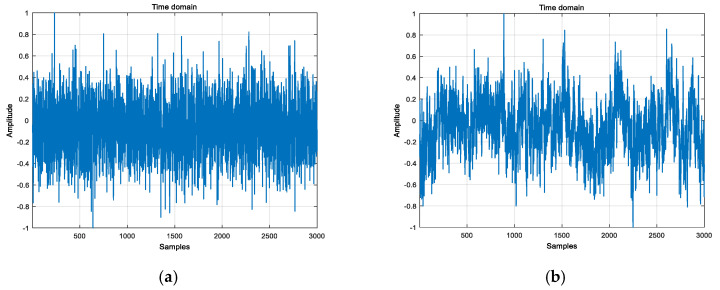
The time waveform for two simulated signals: (**a**) Gaussian white noise, (**b**) 1/f noise.

**Figure 7:**
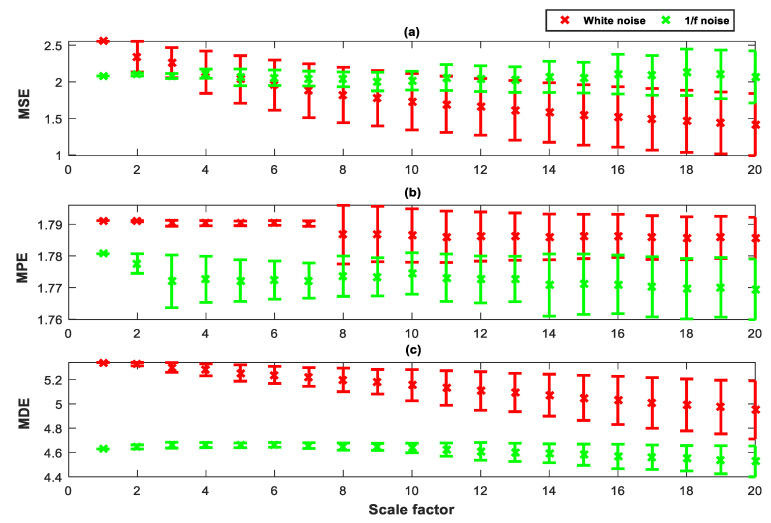
The multi-entropy value of Gaussian white noise and 1/f noise: (**a**) MSE, (**b**) MPE and (**c**) MDE.

To illustrate the advantages of MDE, GWN and 1/f noise data with size of 3000 points are applied to perform the comparison between MSE, MPE, and MDE. Figure 6 shows the time waveform for GWN and 1/f noise. Figure 7 shows the error bars of MSE, MPE, and MDE for two simulated signals. In this simulation, we set m=3, d=1, and the similar tolerance of MSE is set to r=0.15. In [9], the parameters of DE are analyzed in detail, so we selected the parameter of MDE as follows: the number of classes is c=6, and the largest number of scale factor is s=20.

It can be seen from Figure 7 that, on the low scale factor, entropy values of GWN are larger than that of 1/f noise. MSE, MPE and MDE of the GWN correspondingly decrease during the scale factor increasing. This is because the GWN is more irregular than the 1/f noise. In summary, compared with MSE, MPE and MDE, because of the advantage of DE, the MDE calculation result is more stable.

## 3. The Proposed Feature Extraction Method

According to the theoretical analysis of IITD and MDE in Section 2, this paper combines IITD and MDE to present the following feature extraction for ship-radiated noise:

(1) Perform IITD on the five types of ship-radiated noise signals of the training data and decompose signals into a series of ISCs and one monotonic trend component.
(2) Calculate the correlation between ISCs and the original signal, then select the ISCs with large correlation coefficients as the feature parameter.
(3) Calculate their MDE value of the chosen ISCs and set scale factor to 20.
(4) Input feature vectors to SVM to establish the classifier.
(5) For the test dataset, extract their features using steps (1–3), then input the features into classifier for classification and get recognition rates.

**Figure 8:**
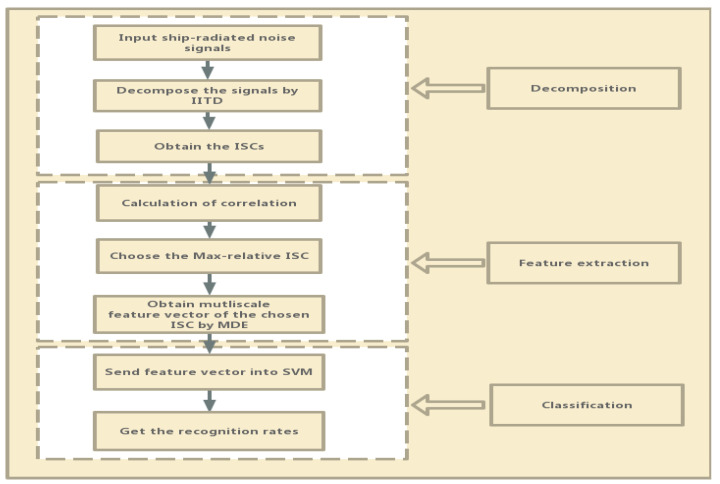
The flowchart of feature extraction of ship-radiated noise based on IITD-MDE.

Figure 8 shows the detailed flowchart of the IITD-MDE.

## 4. Experimental Verification and Analysis

**Figure 9:**
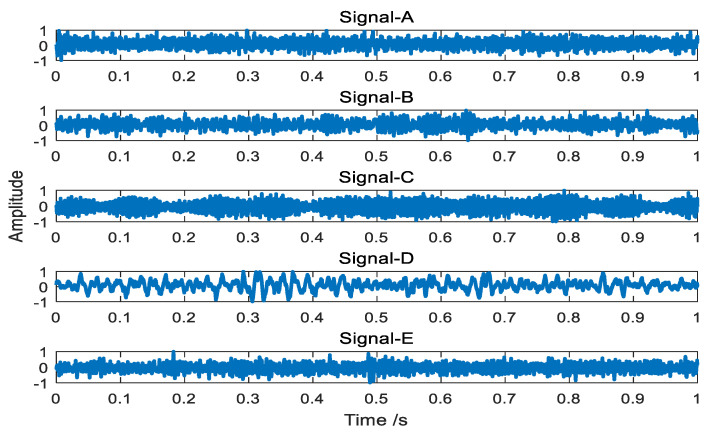
Five types of ship signals.

**Figure 10:**
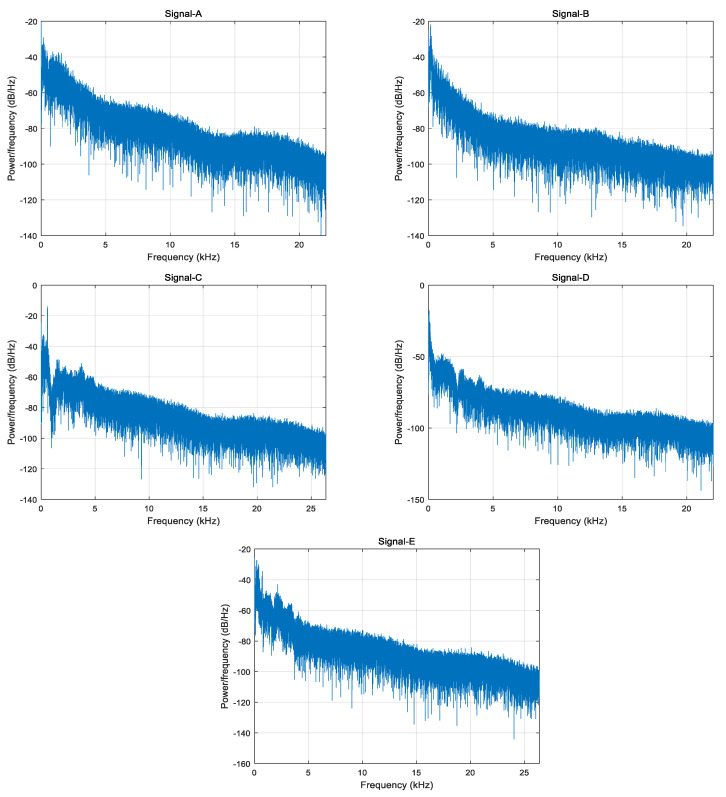
Spectrum analysis.

In order to verify the effectiveness of the proposed method, all data we used are actual ship-radiated noise signals under the same conditions. Five different types of ship-radiated noise signals are selected as sample dataset, which including ferry ship (Signal-A), cruise ship (Signal-B), passenger ship (Signal-C), submarine (Signal-D), and oceanline (Signal-E). The sampling frequency of Signal-A, Signal-B, and Signal-D are 44.1 kHz and the sampling frequency of Signal-C and Signal-E are 52,734 Hz. Figure 9 and Figure 10 respectively shows the time domain waveforms and spectrum analysis of the normalized ship signals.

### 4.1. IITD Decomposition

**Figure 11:**
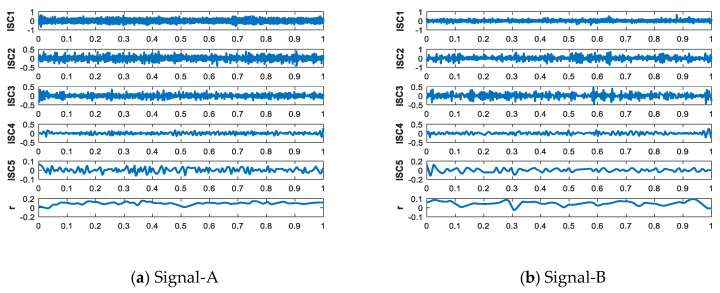

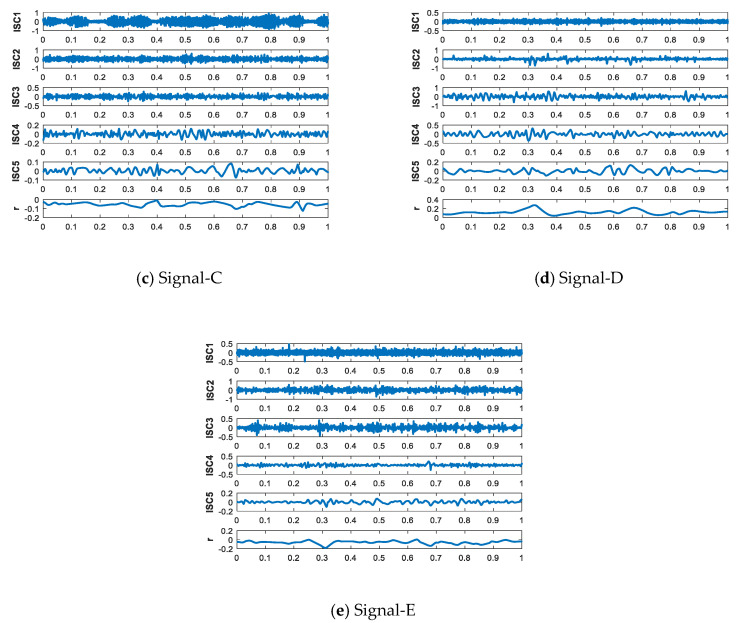
Time domain of decomposed results by IITD.

**Figure 12:**
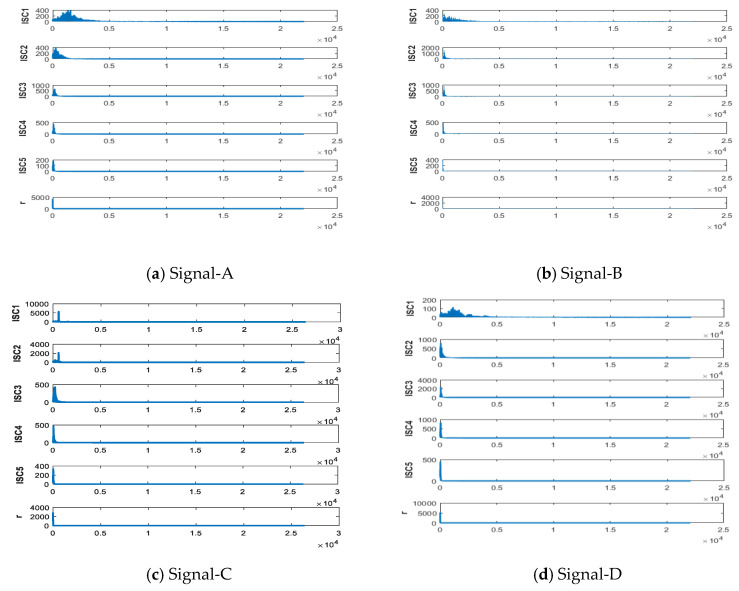

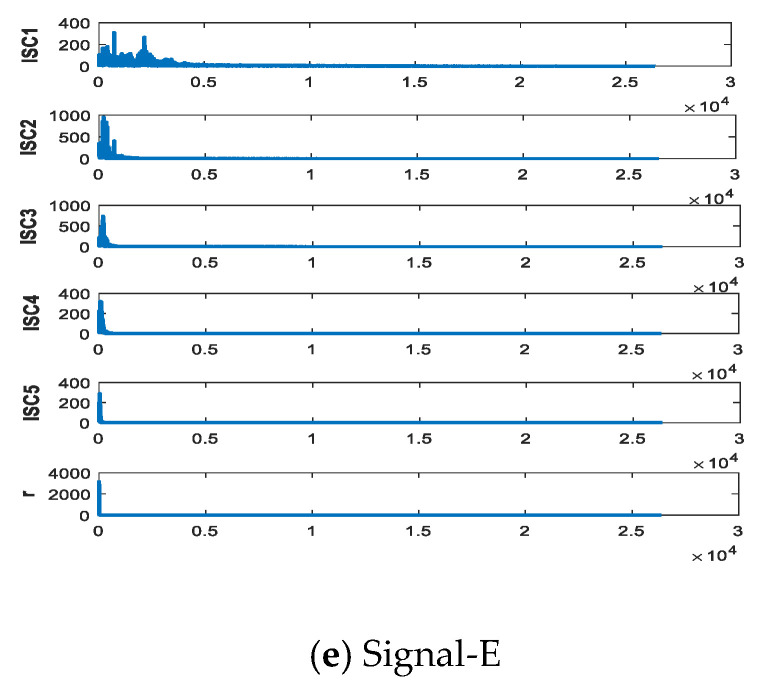
Spectrum of decomposed results by IITD.

IITD is decomposed the five different types of ship-radiated noise, and the results as shown in Figure 11 and Figure 12.

It can be seen from Figure 11 that ship-radiated noise signals can be decomposed of five ISCs and one monotonic trend signal. Figure 12 shows the frequency of the signals are arranged from high frequency to low frequency. The ISCs of different ship-radiated noise signal are different indicate that the complexity of each type of signals are different. Hence, we can use each order component as a feature vector.

### 4.2. ISC Choosen

**Figure 13:**
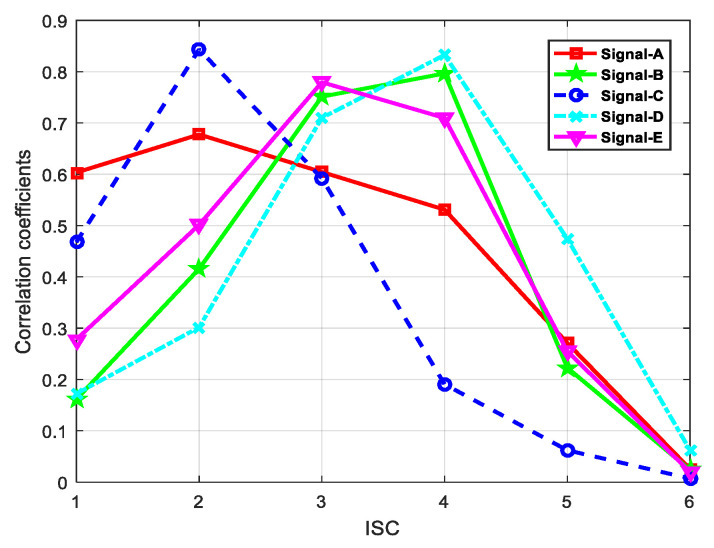
Correlation coefficients of ISCs.

In order to obtain the ISC that contain the major information characteristics of the original signal, we calculated the correlation coefficients between each ISC and the original signal, and results are shown in Figure 13.

Table 1 shows the feature parameters of different ship signals are distributed in different orders, which means these ISCs could represent the effective component. Therefore, the largest correlation coefficient result is selected and analyzed for feature parameter.

### 4.3. Feature Extraction

**Figure 14:**
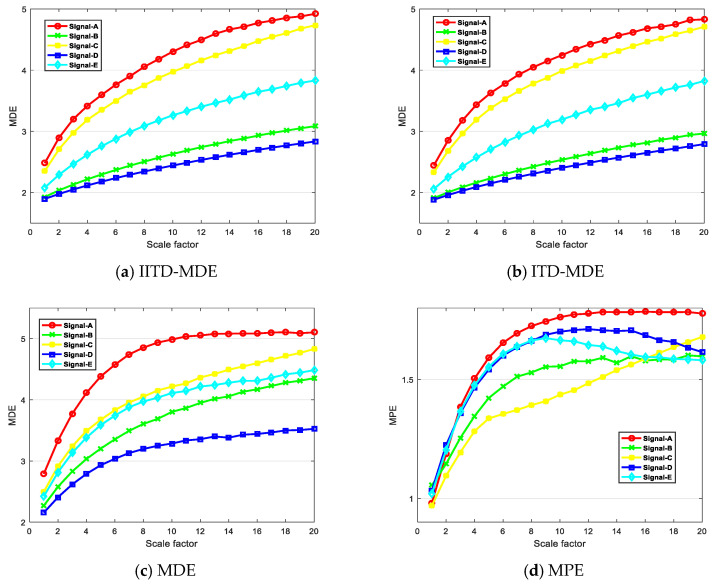
The distribution of the four methods.

**Figure 15:**
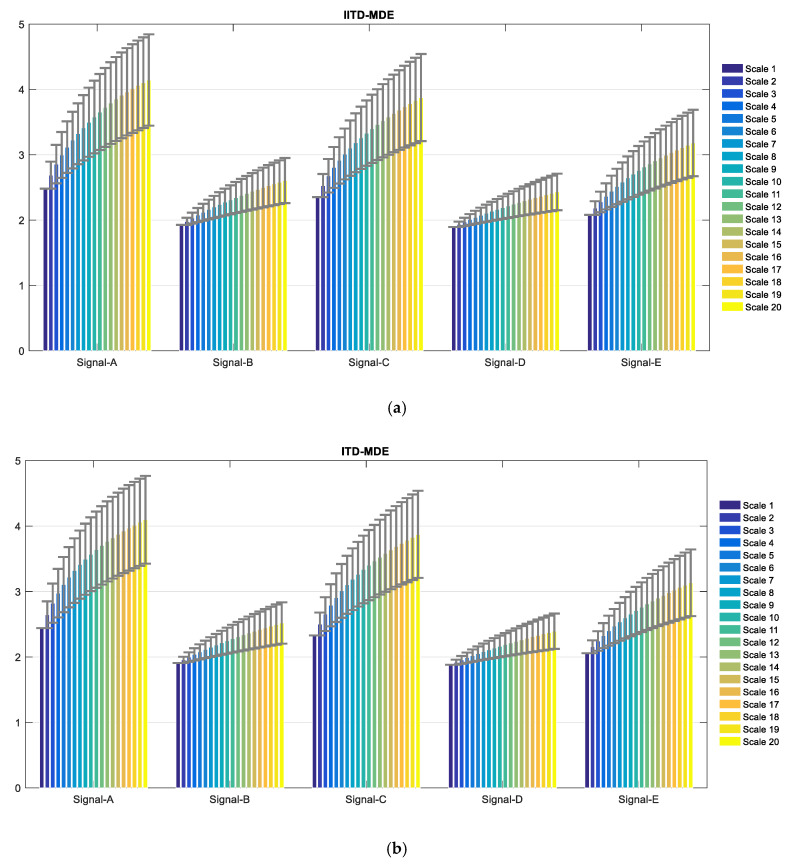
Error bar graph of the methods (**a**) IITD-MDE and (**b**) ITD-MDE.

The proposed technique was utilized to five different types of ship-radiated noise signal. Figure 14a shows the IITD-MDE distribution of ship signals. the abscissa represents the scale factor, and the ordinate represents the feature vector MDE. The results demonstrate that the IITD-MDE value is at the same level for the same ships, but there is an obvious difference for different types of ships. The means and standard deviations of this method are shown in Figure 15a. It can be concluded that the means and standard deviations of the proposed feature extraction method are different, while others are close to each other and the ranges of fluctuations are severely overlapping and non-separable. This indicates that the proposed feature extraction is reliable.

In order to demonstrate the superiority of the IITD-MDE method proposed in this paper, different feature extraction methods are applied to the same dataset, including the ITD-MDE method, the MDE method and the MPE method. The ITD-MDE method results are depicted in Figure 14b. The means and standard deviations of this method are shown in Figure 15b. Compared with Figure 14a, the results demonstrate the overall entropy values are lower than IITD-MDE method. Therefore, the proposed feature extraction method is better to distinguish ship signal. The distribution of MDE method is shown in Figure 14c. It can be seen that the MDE of Signal-A is the largest, and the MDE of Signal-D is the smallest. There is a large overlap between Signal-B, Signal-C, and Signal-E. The distribution of MPE method is shown in Figure 14d. Compared with Figure 14c, the MDE method is smoother and more stable than the MPE method.

### 4.4. Ship Classification

**Figure 16:**
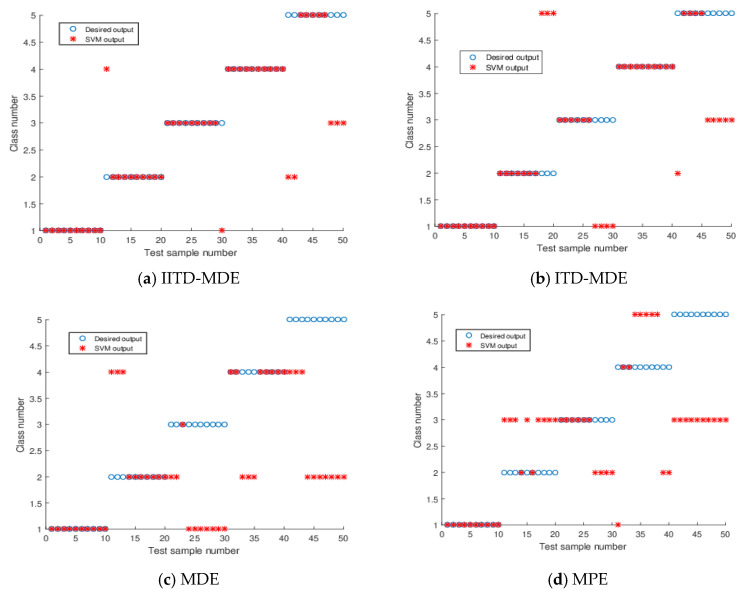
Classification results.

The feature vectors obtained in Section 4.3 are input into SVM [34] for identification and classification of ship-radiated noise. For each type of ship signal, 20 samples have been selected. In this case, 10 samples are used as training set and the remaining 10 samples are used as a test set. In order to further analyze the classification results, the MDE, MPE, and ITD-MDE method were also used to classify ship signal. The classification results are shown in Figure 16, and the recognition accuracies are listed in Table 2. For each type of ship signal, the MPE method is not completely classified correctly, and the classification accuracy is 40%. The MDE method is inferior to the MPE method, and the classification accuracy is 50%. The ITD-MDE method is inferior to the MDE method and classification accuracy is 74%. Compared with the other three methods, the classification accuracy of the proposed method reaches 86%. The results indicate that the proposed method can better classify the five types of ship signals.

## 5. Conclusions

In this paper, we carried out an investigation aimed at gaining a better recognition accuracy of ship-radiated noise signals, a new feature extraction method based on IITD-MDE is present. We also introduced IITD and MDE to quantify the ship-radiated noise signal in this article.

The work done here has following implications. Firstly, we showed that IITD is appropriate approach, compared with ITD and EMD, when dealing with noise signal. We also found that MDE are suitable to quantify the extracted ship-radiated noise feature information, compared with MSE and MPE. Finally, the most consistent method to distinguish the different types of ship-radiated noise signals was IITD-MDE and recognition rate is 86%, compared with ITD-MDE, MDE, and MPE. Hence, the proposed method can extract ship feature and classify effectively.

## Figures and Tables

**Table 1:** Select as a feature parameter.

**Ship Signal**	Signal-A	Signal-B	Signal-C	Signal-D	Signal-E
**Feature Parameter**	ISC2	ISC4	ISC2	ISC4	ISC3

**Table 2:** Different methods for accuracy.

Methods	Accuracy Rate		
	Accuracy	Mean Squared Error	Squared Correlation Coefficient
IITD-MDE	86%	0.56	0.7356
ITD-MDE	74%	1.44	0.4661
MDE	50%	2.32	0.2680
MPE	40%	1.84	0.2386
